# Protonema of the moss *Funaria hygrometrica* can function as a lead (Pb) adsorbent

**DOI:** 10.1371/journal.pone.0189726

**Published:** 2017-12-20

**Authors:** Misao Itouga, Manabu Hayatsu, Mayuko Sato, Yuuri Tsuboi, Yukari Kato, Kiminori Toyooka, Suechika Suzuki, Seiji Nakatsuka, Satoshi Kawakami, Jun Kikuchi, Hitoshi Sakakibara

**Affiliations:** 1 RIKEN Center for Sustainable Resource Science, Tsurumi, Yokohama, Japan; 2 Department of Biological Sciences, Faculty of Science, and Research Institute for Integrated Science, Kanagawa University, Hiratsuka, Japan; 3 DOWA Technology Co., Ltd., Chiyoda, Tokyo, Japan; 4 DOWA Eco-System Co., Ltd., Chiyoda, Tokyo, Japan; 5 Graduate School of Bioagricultural Sciences, Nagoya University, Chikusa, Nagoya, Japan; Massey University, NEW ZEALAND

## Abstract

Water contamination by heavy metals from industrial activities is a serious environmental concern. To mitigate heavy metal toxicity and to recover heavy metals for recycling, biomaterials used in phytoremediation and bio-sorbent filtration have recently drawn renewed attention. The filamentous protonemal cells of the moss *Funaria hygrometrica* can hyperaccumulate lead (Pb) up to 74% of their dry weight when exposed to solutions containing divalent Pb. Energy-dispersive X-ray spectroscopy revealed that Pb is localized to the cell walls, endoplasmic reticulum-like membrane structures, and chloroplast thylakoids, suggesting that multiple Pb retention mechanisms are operating in living *F*. *hygrometrica*. The main Pb-accumulating compartment was the cell wall, and prepared cell-wall fractions could also adsorb Pb. Nuclear magnetic resonance analysis showed that polysaccharides composed of polygalacturonic acid and cellulose probably serve as the most effective Pb-binding components. The adsorption abilities were retained throughout a wide range of pH values, and bound Pb was not desorbed under conditions of high ionic strength. In addition, the moss is highly tolerant to Pb. These results suggest that the moss *F*. *hygrometrica* could be a useful tool for the mitigation of Pb-toxicity in wastewater.

## Introduction

Water is essential for all living organisms on earth. Humans do not only ingest water, but use it also for agriculture and industrial activities. Water contamination with heavy metals from human industrial activities is a serious environmental concern. If polluted water enters drinking and agricultural water systems, heavy metals may cause serious toxicity to organisms [1]. To remove heavy metals from contaminated water, various methods and remediation materials have been developed and are widely used in current industrial procedures (e.g. chemical sedimentation, electro deposition, activated charcoal, ion-exchange resins, chelating resins) [2]. However, these technologies often require materials derived from fossil resources, consume substantial amounts of energy, and emit CO_2_. Thus, the development of alternative, environment-friendly remediation technologies based on CO_2_-fixing organisms would be an important step towards more sustainable industrial processes.

Recently, biomaterials used in phytoremediation and bio-sorbent filtration have enjoyed renewed attention. Phytofiltration and rhizofiltration technologies [3, 4] using living plants or non-living plant residues have been evaluated in various water cleaning systems. For instance, rhizofiltration using sunflower (*Helianthus annuus* L.) and bean (*Phaseolus vulgaris* L. var. *vulgaris*) roots can remove uranium (U) from groundwater [5]. Agricultural bio-wastes such as banana skins, green tea waste, oak leaves, walnut shells, peanut shells, and rice husks are all effective in removing chromium (Cr) from wastewater [6]. Recent studies have also shown that modified agricultural bio-wastes, such as orange peels and apple residues, have a high adsorption capacity for heavy metals in aqueous solution [7, 8]. Although these classical, recycling-based approaches remain important for phyto-remediation, it is highly desirable to develop new bio-materials that can be produced from non-fossil resources.

Certain bryophytes (mosses and liverworts) occasionally are found in environments with immoderate soil pH values and/or high metal concentrations because these plants can grow under such harsh conditions. These bryophytes play a role as pioneer plant species in the restoration of soil fertility. Since bryophytes are non-vascular plants and do not have a root system, water and minerals are absorbed by the entire body and their growth and development are readily affected by the surrounding fluid conditions. Thus, certain bryophytes are hypothesized to have specialized mechanisms to detoxify or sequester negative chemical factors. In addition, although the growth rate of moss leafy gametophytes is generally lower than for typical vascular plants, protonemal cells are capable of proliferating as rapidly as filamentous algae in liquid culture conditions [9].

We became interested in the ability of bryophytes to tolerate and remove heavy metals from aqueous solutions. About 400 plant species are listed as prospective resources that could be used for metal remediation, and about 30 of these plants are bryophytes (mosses and liverworts) [10]. Several recent studies have characterized the metal specificity of specific bryophytes and have demonstrated the possibility of using bryophytes in phyto-remediation. For instance, although *Scopelophila cataractae* (Mitt.) Broth. and *Scopelophila ligulata* (Spruce) Spruce are known as typical copper (Cu) accumulating mosses [11–13], their metal-adsorption capabilities differ significantly. *S*. *cataractae* has a high Cu-adsorption capacity, whereas *S*. *ligulata* has a particularly high adsorption capacity for iron [14]. When these mosses were used to mitigate Cu-toxicity in cultured rice, the effects of *S*. *cataractae* proved superior to those of *S*. *ligulata* as evaluated by the photosynthetic rates and genome-wide expression profiles of rice leaves [15]. A comparative study with *Physcomitrella patens*, *Polytrichum formosum*, and *S*. *cataractae* also suggested that *S*. *cataractae* could be used to treat Cu-polluted wastewater [16]. Collectively, these studies hint at the potential for using bryophytes as a new bio-material in the mitigation of metal-polluted wastewater.

In this study, we focused on *Funaria hygrometrica*, a moss that is often seen growing on metal-enriched substrates, such as mine sites contaminated with Cu, zinc (Zn), lead (Pb) and other heavy metals [17, 18], or in places recovering from wild fires [19, 20]. These habitats led us to hypothesize that *F*. *hygrometrica* might have a special ability for metal tolerance and accumulation, but detailed characterization of this moss has not been previously reported. Here, we report that *F*. *hygrometrica* adsorbs Pb to extraordinary levels when protonema are exposed to solutions containing these ions. We characterized the cellular localization, metal specificities, cell-wall components, and effects of chemical factors on adsorption and desorption. Our results suggest that using the moss *F*. *hygrometrica* to mitigate Pb toxicity could help develop sustainable water cleaning systems.

## Materials and methods

### Moss sampling and spore sowing

*Funaria hygrometrica* was collected from reclaimed land in Omuta City, Fukuoka, Japan (130°23′E, 33°1′ N), in April 2003. The spores were sown on a modified Knop’s-agar medium: 10 mM KNO_3_, 1 mM MgSO_4_, 2 mM KH_2_PO_4_, 10 mM CaCl_2_, 45 μM FeSO_4_, 1.6 μM MnSO_4_, 10 μM H_3_BO_3_, 0.2 μM ZnSO_4_, 0.2 μM KI, 0.1 μM Na_2_MoO_4_, 0.2 μM CuSO_4_, 0.2 μM CoCl_2_, 5 mM (NH_4_)_2_C_4_H_4_O_6_, pH 5.5, and 1% (w/v) agar in plastic Petri dishes. Cellophane (PL-#300, Futamura Chemical Industries Co., Ltd., Japan) washed with 5 mM EDTA·4Na and MilliQ water (MQW) was placed on the media before sowing. Protonemal cells were grown under fluorescent light at 80 μmol m^-2^ s^-1^ with a 16 h light / 8 h dark cycle at 23°C.

### Establishment of protonemal suspension cultures

Approximately 50 mg fresh weight of *F*. *hygrometrica* protonemal cells grown on agar were collected and suspended in modified Knop’s liquid medium using a Polytron homogenizer (PT-MR2100, Kinematica, Switzerland) operated at minimum speed for 15 sec. The suspension cells were inoculated into 0.5 L liquid medium in a culture bottle, and cultured with aeration at 1000 mL min^-1^ under fluorescent lights. The growth rate of the culture was examined by measuring gain of the dry weight of cells at 5 and 8 days after inoculation.

### Morphological observation of filamentous protonemal cells

Cultured *F*. *hygrometrica* protonemal cells were collected by filtration through a suction funnel fitted with a glass fiber filter (GS25, Advantec). The cells were freeze-dried and observed with a scanning electron microscope (SEM) (TM3000, Hitachi).

### Adsorption test for 15 metal elements

Two-week-old cultured protonemal cells of *F*. *hygrometrica* were used as adsorbent materials. Twenty mL of the suspension culture was poured into a glass column (Fh-column; 10 mm inner diameter × 100 mm length). The Fh-column was washed and equilibrated with MQW using a peristaltic pump at a flow rate of ca. 12.5 mL h^-1^ for 18 h. The test solutions were prepared by dissolving the following reagents in MQW to 100 μM final concentration. The reagents and pH values of the solutions were as follows: Li, LiCl (pH 4.7); Al, AlCl_3_, (pH 4.0); Cr, CrCl_2_ (pH 3.4); Mn, MnCl_2_ (pH 4.1); Co, CoCl_2_ (pH 3.9); Ni, NiCl_2_, (pH 4.1); Zn, ZnCl_2_, (pH 4.5); Mo, MoCl_5_, (pH 2.2); Pt, PtCl_4_, (pH 2.2); Tl, TlCl, (pH 6.1); Pb, PbCl_2_ (pH 4.8). The test solutions for Ag and Au were prepared by dissolving AgCl and AuCl in 1 mM HCl (pH 3.0) to final concentrations of 100 μM. Test solutions of Se and Y were prepared by diluting commercial 1000 mg L^-1^ standard solutions (Se, 192–13861; Y, 250–00121, Wako Pure Chemical Industries, Japan) with MQW to 50 mg L^-1^ (ie. Se, 63 μM; Y, 56 μM). The pH values of the Se and Y solutions were 3.2 and 2.2, respectively. Each test solution containing metal ions was loaded onto the Fh-column for 22 h, followed by washing the column with MQW for 8 h. Completeness of washing was checked in every experiment. The filtrate was collected in increments of approximately 5 mL using a fraction collector. An outline of this procedure is given in S1 Fig.

### Metal determination and quantification

To analyze the Fh-column filtrates, each solution was directly injected into an inductively coupled plasma mass spectrometer (ICP-MS; Perkin Elmer Elan6100DRC). For analysis of moss plant material, the protonemal cells were dried for 3 days at 60°C and predigested with 5 mL aqua regia (HNO_3_:HCl = 1:3) overnight at room temperature. Thereafter, the organic compounds were totally decomposed by wet-ashing using a microwave sample preparation system (Perkin Elmer MultiWave-3000). The volume of the digested samples was adjusted to 50 mL with MQW, and the solution was filtered through 5B filter paper (Advantec, Tokyo, Japan). For ICP-MS analysis, a portion of the filtered samples was diluted appropriately with MQW.

### Electron microscopic analysis

*F*. *hygrometrica* cells used for Pb-adsorption tests and non-treated control cells were fixed in 2.5% glutaraldehyde in 50 mM K-phosphate buffer (pH 7.4) for 1.5 h. The sample was washed three times with 50 mM K-phosphate buffer (pH 7.4) and postfixed in 1% OsO_4_ dissolved in the same buffer at room temperature for 1 h. The fixed samples were dehydrated through a graded methanol series (12.5, 25, 50, 70, 80, 90, 95, and 100%) and embedded in Epon812 resin (TAAB, Berkshire, UK). Ultrathin sections (80 nm) were obtained by cutting with diamond knives on an Ultracut UCT ultramicrotome (Leica, Vienna, Austria) and were transferred to formvar-coated grids. The sections were stained with 4% uranyl acetate for 12 min and examined with a transmission electron microscope (JEM-1011; JEOL, Tokyo, Japan). Images were acquired using a Gatan DualView CCD camera and Gatan Digital Micrograph software.

For metal identification, *F*. *hygrometrica* cells used for Pb-adsorption tests were examined using a TEM-EDX spectroscopy system [JEM-1230 electron microscope with a MiniCup/EX-14033JTP energy dispersive X-ray (EDX) microanalyzer (JEOL, Tokyo, Japan)] [21]. Section thickness was increased to150 nm for EDX to get as much of signal as possible, and uranium staining was omitted to cut X-ray signal off originated from heavy metals except for Pb and to find dense particle more easily.

### Distribution of Pb between cell walls and other components

After *F*. *hygrometrica* protonemal cells adsorbed Pb, the cells were freeze-dried and divided into two parts: one portion was used to prepare a cell-wall fraction (CWF) and the other portion served as the total cell fraction (TC). To prepare the CWF, the cells were suspended in aqueous 80% (v/v) ethanol and homogenized using a pestle and a mixer (Pellet Pestle^®^ Cordless Motor, Kimble Chase, USA). After centrifugation at 1,200 × *g* for 5 min, the pellet was washed in a sequence of 80% ethanol, 95% ethanol, 99.5% ethanol, chloroform:methanol (1:1), and acetone. The alcohol-insoluble residues were dried in air and analyzed as the cell-wall fraction as previously described by Matsunaga et al. [22]. The Pb contents of the CWF ([Pb]_CWF_) and TC ([Pb]_TC_) preparations were determined by ICP-MS. To determine the cell-wall proportion in *F*. *hygrometrica* protonemal cells (CWF/TC), *F*. *hygrometrica* protonemal suspension cells were washed with MQW, freeze-dried, and weighed. Then, the cell-wall fraction was prepared as described above and weighed. Distributions of Pb in the cell walls were estimated according to:

$$\text{Pb}\;\text{content}\;\text{in}\;\text{cell}\;\text{walls}\;({\left[\text{Pb}\right]}_{\text{CW}})={\left[\text{Pb}\right]}_{\text{CWF}}\times \text{CWF}/\text{TC}$$

$$\text{Distribution}\;(\%)\;\text{of}\;\text{Pb}\;\text{in}\;\text{cell}\;\text{walls}={\left[\text{Pb}\right]}_{\text{CW}}/{\left[\text{Pb}\right]}_{\text{TC}}\times 100$$

### Pb-adsorption to the prepared cell-wall fraction

*F*. *hygrometrica* protonemal cells were cultured for 10 days on the modified Knop’s-agar medium, harvested and freeze-dried. Cell walls were extracted by homogenizing the dried samples in 50 mM Na-phosphate buffer (pH 6.5), collecting the cell walls by centrifugation, followed by washing several times with 50 mM Na-phosphate buffer (pH 6.5) until the wash solution was visibly colorless. Finally, cell walls were sequentially washed with acetone, a water/chloroform/methanol mixture (5:6:3, v/v/v) and freeze-dried. The prepared cell-wall fractions were left to stand for 4 h in glass bottles filled with 1 mM PbCl_2_, Pb(NO_3_)_2_, or MQW (Mock). After washing with MQW, bound Pb was detected as a red lead-rhodizonate complex visualized by staining with 3 mM sodium rhodizonic acid and 100 mM tartaric acid (pH 2.8) on a slide glass or by X-ray analysis with an X-ray analyzer (XGT-5000, Horiba, Japan).

### NMR analysis of cell-wall components

Sample preparation for solubilized cell-wall components from *F*. *hygrometrica* cells was like that described in previously published reports for land plants and macroalgae [23–25]. Sample solutions (4:1 dimethyl sulphoxide (DMSO)-d_6_:pyridine-d_5_) were transferred into 5-mm *ϕ* NMR tubes and subjected to NMR analysis. The temperature of all NMR samples was maintained at 298 K. The chemical shifts were referenced to the methyl group of DMSO–d_6_ at ^13^C = 40.03 ppm and ^1^H = 2.582 ppm, respectively. Two-dimensional ^1^H-^13^C Hetero-nuclear Single Quantum Coherence (HSQC) spectra were collected using essentially similar conditions to our previous reports [26, 27], and free-induction decay data were processed as in our previous reports [28, 29]. 1,3-beta-Glucan (Curdlan, product no 032–09902, Wako Pure Chemical, Osaka, Japan), pectin (from Citrus, 164–00552, Wako Pure Chemical), polygalacturonic acid (PGA; product no 102711, MP Biomedicals, CA, USA) and cellooligosaccharides (cellobiose, product no 400398; cellotriose, 400400; cellotetraose, 400402; cellopentaose, 400404; cellohexaose, 400406; Seikagaku Corporation, Tokyo, Japan) were used as standard compounds. We used cellooligosaccharides because cellulose cannot be dissolved in the DMSO/pyridine solvent.

### Effects of pH on metal adsorption

The test solutions were prepared by diluting the commercial standard solutions (Wako Pure Chemical Industries) with MQW to concentrations of 5 mg L^-1^ (Au and Pt-group metals) or 10 mg L^-1^ (other metals). Five g (wet weight) of *F*. *hygrometrica* protonemal cells were suspended in 250 mL of the test solutions containing the tested metals at various pH values, and the suspensions were incubated for 5 h with shaking at 100 r.p.m. During the incubation, the pH was occasionally checked and adjusted within ±0.15 of the initial value with diluted HCl or NaOH. After incubation, the filtrate was recovered and analyzed using an inductively coupled plasma atomic emission spectrometer (ICP-AES; SPS5100, SII NanoTechnology, Chiba, Japan).

### Effects of ionic strength on Pb desorption

Pb-loaded *F*. *hygrometrica* protonemal cells were prepared by incubation with 100 mg L^-1^ Pb (pH 5.0) for 24 h, followed by washing with MQW. The resulting Pb concentration of the protonemal cells was 4.02 mg g^-1^ wet weight. Five g (wet weight) of the Pb-adsorbed cells was suspended in 250 mL of MQW, the pH was adjusted to 5.0 with HCl, and the ionic strengths of the suspensions were adjusted by adding an aqueous solution of NaCl. The suspensions were incubated for 5 h with shaking at 100 r.p.m. After incubation, the samples were filtered and the recovered filtrate was subjected to ICP-MS (Agilent 7500, Agilent Technologies).

## Results

### *F*. *hygrometrica* protonemal suspension cultures

**Fig 1:**
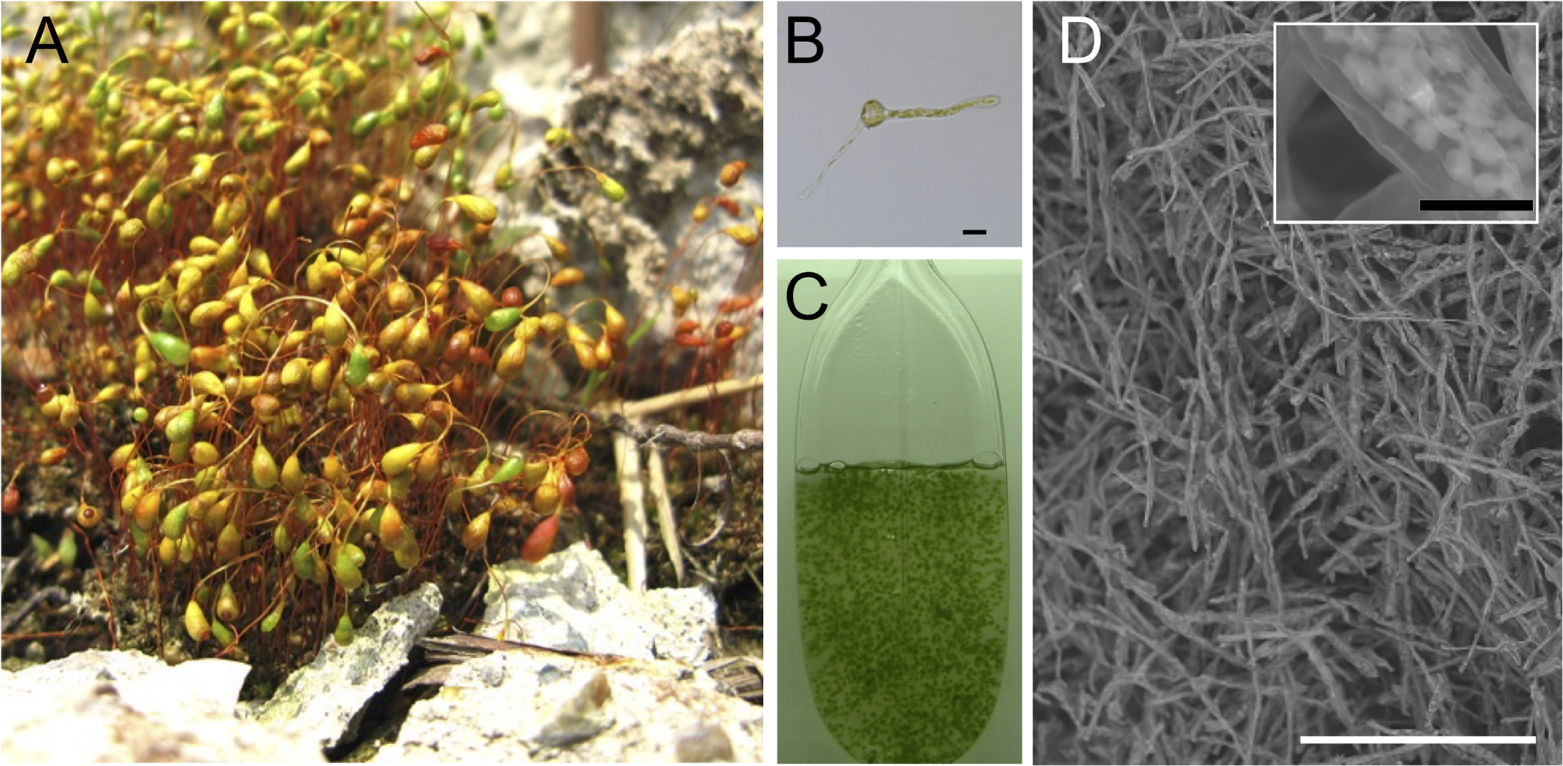
*Funaria hygrometrica* Hedw. (A) Colony of gametophytes bearing bright yellowish mature sporophytes. (B) Spore germination at 48 hours after sowing. Scale bar = 20 μm. (C) Suspension culture in a 0.5 L bottle. (D) SEM photograph of a freeze-dried cell pellet. Bright spots in the inset represent chloroplasts. Scale bar = 250 μm. A magnified view of the cells is shown in the inset. Scale bar = 10 μm.

We collected *F*. *hygrometrica* Hedw. on a landfill area in Omuta City, Japan, on Apr. 16, 2003 (Fig 1A). To evaluate the ability of *F*. *hygrometrica* to adsorb various metal ions, it was essential to work with uniform material. Thus, we collected sporophytes, allowed the spores to germinate on agar plates (Fig 1B), and established a suspension culture of protonemal cells derived from a single spore (Fig 1C). When the protonemal cells were cultured in modified Knop’s liquid medium, the constant growth rate in the exponential phase (μ_e_) was 0.144 g dry weight L^-1^ d^-1^ and the final yield was ca. 0.57 g dry weight L^-1^. The collected protonemal cells at the late stationary phase (i.e. 2 weeks) were used as metal adsorbents. Observation of the fine structure of collected *F*. *hygrometrica* protonemal cells with SEM showed that the filamentous protonemal cells were intertangled (Fig 1D).

### Hyper-accumulation of Pb and other metal ions by *F*. *hygrometrica*

To examine the capacity of *F*. *hygrometrica* to adsorb industrially important metal ions, we prepared protonema-packed small columns and loaded 15 metal or metalloid ion solutions onto the columns (S1A Fig). To establish the maximum adsorption capacities, thirty-five-column volumes of metal solutions were loaded. After extensive washing, the metal contents were analyzed using an inductively coupled plasma mass spectrometer (ICP-MS) (Table 1). The protonemal cells accumulated remarkably high levels of some metals: Pb was efficiently adsorbed to the breakthrough point (S1B Fig) and accumulated up to 74.1% on a dry weight base. Au was also highly accumulated up to 11.3% (Table 1, S1B Fig), but Li did not accumulate. The order of maximum adsorption capacity was Pb>Au>Cr>Tl>Pt>Co>Mn>Mo>Ni>Al>Ag>Zn>Y>Se>Li. Since the capacity for Pb accumulation was remarkably high, we focused on Pb adsorption by *F*. *hygrometrica* in our further studies.

**Table 1:** Maximum adsorption capacity for 15 elements in the moss *Funaria hygrometrica*.

Element name	Symbol	Maximum adsorption capacity^a^
		[%]
Lithium	Li	0.0
Aluminum	Al	0.9
Chromium	Cr	6.5
Manganese	Mn	1.3
Cobalt	Co	2.5
Nickel	Ni	0.9
Zinc	Zn	0.5
Selenium	Se	0.1
Yttrium	Y	0.2
Molybdenum	Mo	1.2
Silver	Ag	0.7
Platinum	Pt	4.2
Gold	Au	11.3
Thallium	Tl	5.5
Lead	Pb	74.1

^*a*^ Values are percentages on a dry weight basis.

### Cellular localization of accumulated Pb

**Fig 2:**
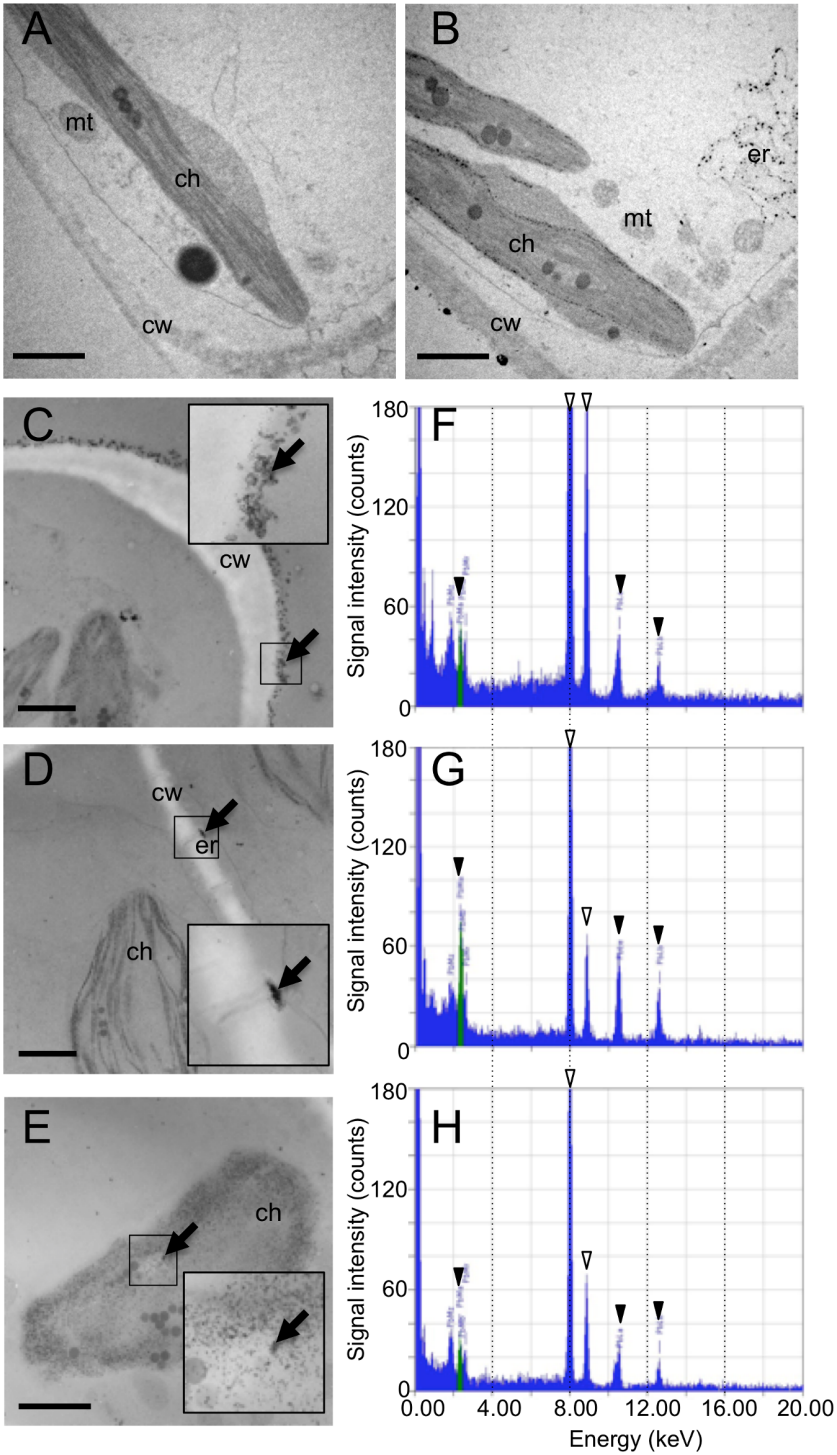
TEM-EDX analysis of *F*. *hygrometrica* protonemal cells. (A) Control cell. (B to E) Cells treated with 0.1 mM PbCl_2_. The thickness of (A) and (B) is 80 nm, and that of (C) to (E) is 150 nm. (C), (D), and (E) are views focusing on the cell wall, endoplasmic reticulum (plasmodesmata), and chloroplast, respectively. Arrows indicate the X-ray analytical areas. Magnified views of the analyzed areas are shown in the inset. cw, cell wall, ch, chloroplast, er, endoplasmic reticulum, mt, mitochondrion. Scale bars: 1 μm. (F, G, H) Energy-disperse spectroscopic spectra. (F), (G), and (H) show results of the analyses of (C), (D), and (E), respectively. Peaks marked by closed arrowheads were used for Pb identification: 2.35(Mα), 10.55(Lα1) and 10.45(Lα2), and 12.61(Lβ1) and 12.62(Lβ2) keV. Peaks highlighted by open arrowheads originated from the Cu in the sample-holding grid (8.05(Kα1) and 8.91(Kβ) keV).

To obtain insights into the mechanism for Pb accumulation, we fixed the *F*. *hygrometrica* protonema used in the column tests, and analyzed these materials with a transmission electron microscope-linked energy-dispersive X-ray (TEM-EDX) microanalysis unit. When the cells were treated with Pb, the cell walls became significantly thicker than the non-treated control (S2 Fig), and high-density particles were detected in cell walls, endoplasmic reticulum (ER)-like membrane structures, and chloroplast thylakoids (Fig 2A–2E). X-ray microanalysis showed that typical Pb signals originated from the high-density particles (Fig 2F–2H). These results suggested that living *F*. *hygrometrica* accumulated Pb at multiple sites.

To examine the distribution of Pb between the cell wall and other compartments, we prepared cell-wall fractions from the Pb-treated *F*. *hygrometrica* protonemal cells and analyzed their Pb content. We found that most of the Pb (88.7%) was present in the cell-wall fraction (S1 Table). These results suggest that the main Pb-accumulating cellular compartment is the cell wall.

### Adsorption of Pb to a prepared cell-wall fraction of *F*. *hygrometrica*

**Fig 3:**
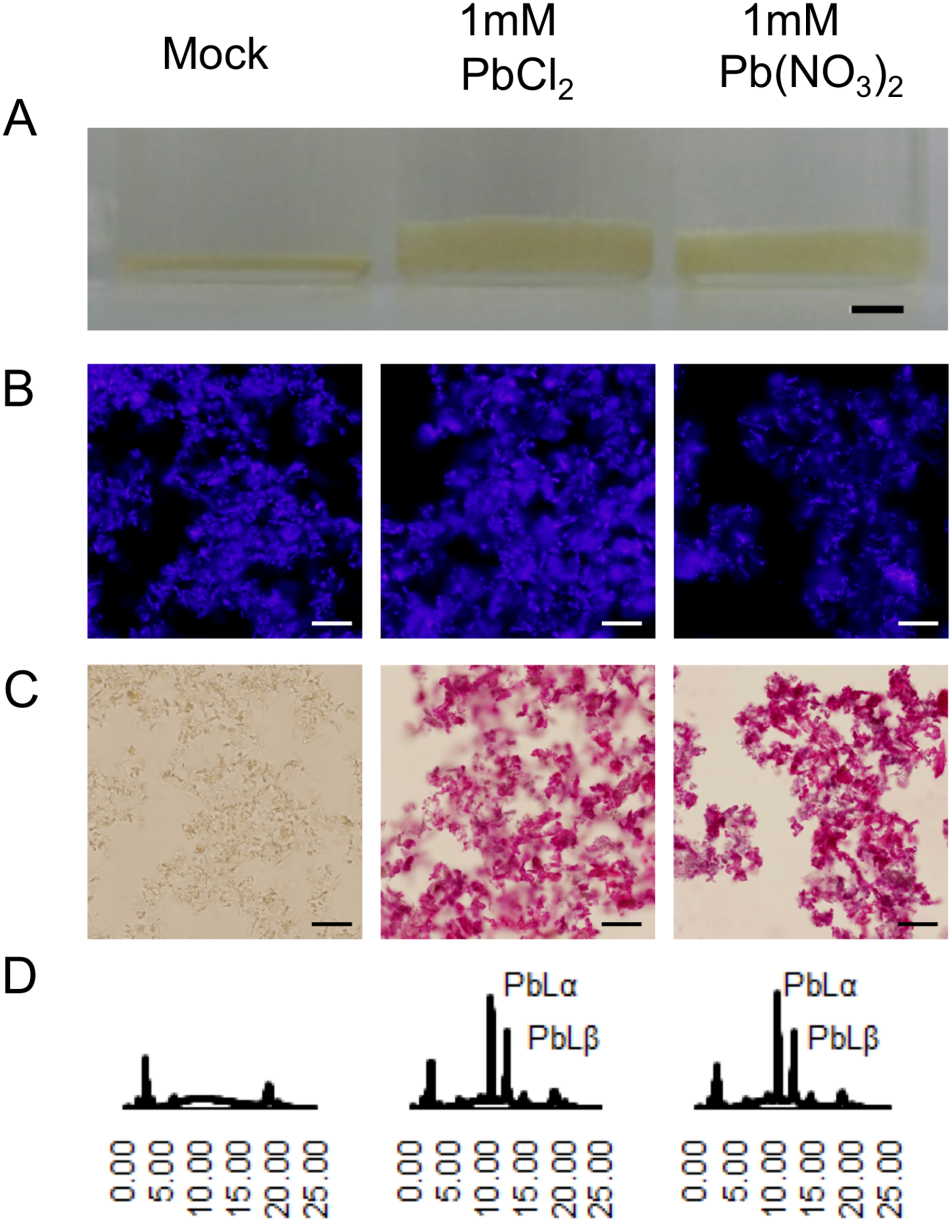
Adsorption of Pb to prepared a cell-wall fraction of *F*. *hygrometrica*. Aliquots of a cell-wall (CW) fraction were suspended in MQW (Mock), 1 mM PbCl_2_, or 1 mM Pb(NO_3_)_2_. After washing with MQW, Pb was detected with rhodizonic acid staining and X-ray analysis. (A) Precipitated CW fractions in bottles after treatment. (B) Autiofluorescence image of rhodizonic acid-stained CW fraction observed with Bio Imaging Navigator with a fluorescence filter (FSX100/U-MNUA2, OLYMPUS, Japan). (C) Observation with bright field mode. (D) Spectra of X-ray analysis with an X-ray analyzer. Background peaks in mock treatment are derived from rhodizonic acid. Scale bars, 3 mm in (A), 100 μm in (B) and (C).

We next examined the adsorption of Pb to a prepared cell-wall fraction of *F*. *hygrometrica*. A Pb-free cell-wall fraction was prepared from *F*. *hygrometrica* protonema, and aliquots of the preparation were treated with 1 mM PbCl_2_ or Pb(NO_3_)_2_. After washing, the bound Pb was detected by staining with rhodizonic acid followed by X-ray analysis. In both Pb-treatments, the cell-wall fraction was strongly stained with rhodizonic acid, and typical Pb signals were detected in the X-ray analysis (Fig 3B–3D), indicating that the *F*. *hygrometrica* cell-wall fraction can adsorb Pb. Notably, Pb-treated cell-wall fractions were swollen compared to those receiving the mock treatment as observed by TEM analysis (Fig 3A and S2 Fig).

### Characterization of cell-wall components by two-dimensional nuclear magnetic resonance

**Fig 4:**
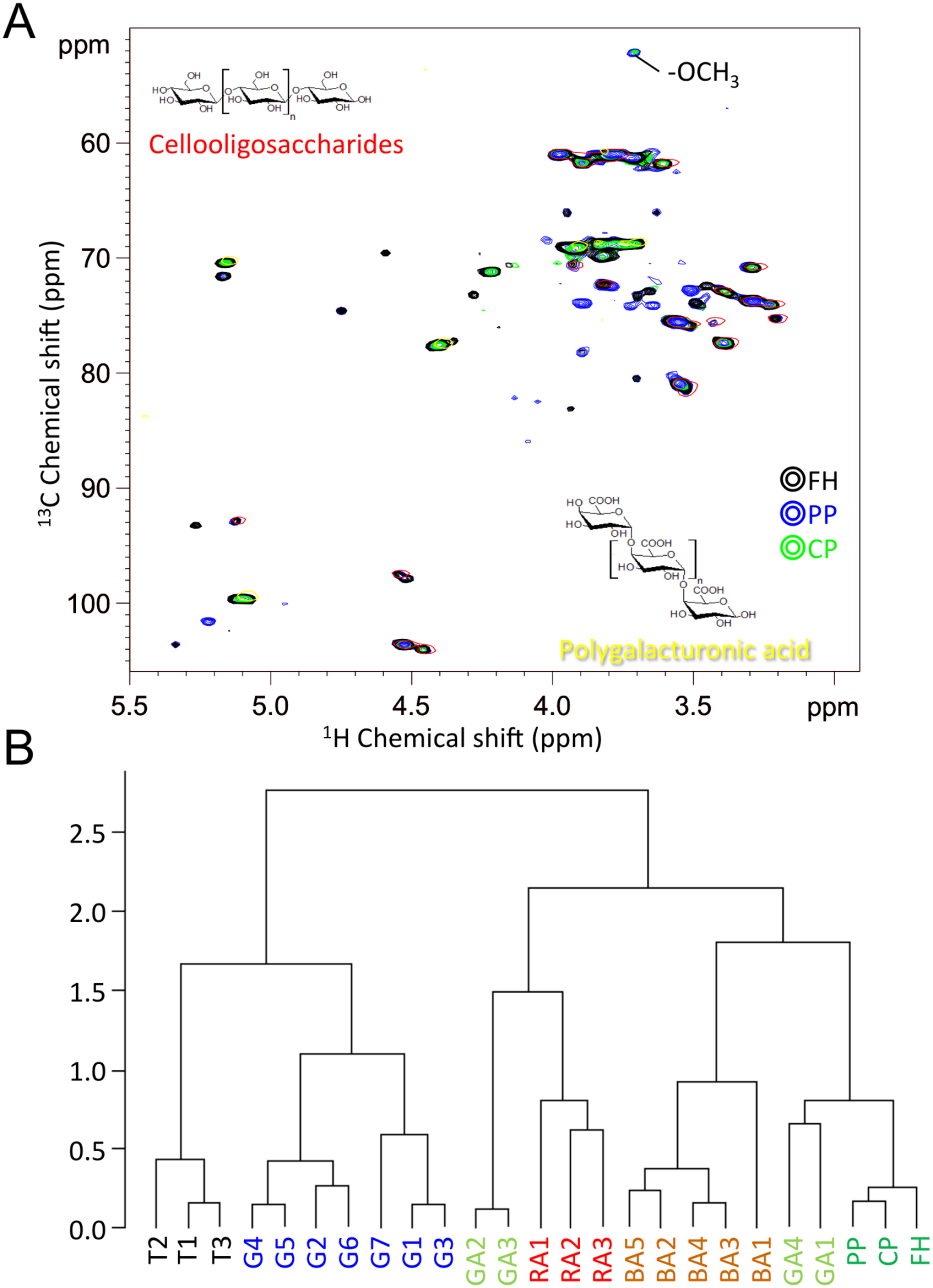
NMR analysis of cell-wall fraction of moss, *F*. *hygrometrica*. (A) ^1^H-^13^C HSQC analysis. Expanded polysaccharide regions of ^1^H-^13^C HSQC spectra from individually characterized commercial reagents, cellooligosaccharides (red) and PGA(yellow), were overlaid on the spectra obtained from the cell-wall fraction of *F*. *hygrometrica* (black), *Physcomitrella patens* (blue), and *Ceratodon purpureus* (yellow-green) (B) Comparison of cell-wall fractions based on hierarchial component analysis of ^1^H-^13^C HSQC spectra. Comparison of cell-wall fraction prepared from land plants, aquatic plants (seeweeds), and *F*. *hygrometrica* based on HCA of their ^1^H-^13^C HSQC spectra. **Trees**: T1, *Castanopsis sieboldii*; T2, *Crypromeria japonica*; T3, *Populus*. **Grasses**: G1, *Erianthus*; G2, *Pennisetum americanum*; G3, *Panicum maximum Jacq*.; G4, *Brachypodium*; G5, *Oryza sativa*; G6, *Triticum aestivum*; G7, *Arabidopsis thaliana*. **Brown algae**: BA1, *Ishige okamurae*; BA2, *Sargassum micracanthum*; BA3, *Sargassum ringgoldianum*; BA4, *Sargassum hemiphyllum*; BA5, *Sargassum patens*. **Green algae**: GA1, *Ulva pertusa*; GA2, *Codium subtubulosum*; GA3, *Codium fragile*. Red algae: RA1, *Gelidium elegans*; RA2, *Ahnfeltiopsis flabelliformis*; RA3, *Prionitis divaricata*. **Bryophytes**: PP, *Physcomitrella patens*; CP, *Ceratodon purpureus*; FH, *Funaria hygrometrica*.

To obtain information about the chemical components of *F*. *hygrometrica* cell walls, the cell-wall components were characterized by two-dimensional nuclear magnetic resonance (NMR). We also prepared cell-wall fractions of two mosses, *Physcomitrella patens* and *Ceratodon purpureus*, for reference. Typical hetero-nuclear single quantum coherence (HSQC) spectra are shown in Fig 4A. In all three moss species, polysaccharide signals predominated in the spectra, including correlations in the chemical shift range of δC/δH 60‒80/3.0‒4.4 ppm, whereas the anomeric correlations in the range of δC/δH 90‒110/4.5‒5.5 ppm were well-resolved. Among the species, the spectral patterns were similar, but not identical. Initially, we attempted to identify or annotate using our chemical shift database of cell-wall components [30, 31]; however, it was difficult to find matched chemical shifts due to the limited cell-wall component data from land plants. Therefore, we compared chemical shifts for several individual commercial reagents, 1,3-beta-glucan, pectin, polygalacturonic acid (PGA) and cellooligosaccharides, that could be assumed to be cell-wall components of bryophytes. A large part of the observed signals from the bryophytes overlapped with PGA and cellooligosaccharides, suggesting that the major cell-wall components adsorbing Pb are PGA and cellulose.

To find the characteristic feature for Pb adsorption by *F*. *hygrometrica* cell walls, we compared our data with previously reported spectral data prepared from land plants and seaweeds [24, 26] and bryophytes *P*. *patens* and *C*. *purpureus*. Hierarchical clustering analysis of normalized HSQC spectra showed that land plants and seaweeds were clustered by separation at the first branch, and bryophytes including *F*. *hygrometrica* were clustered in another branch (Fig 4B). Among members of the clade that includes seaweeds, bryophytes grouped at the edge of the cluster, and *F*. *hygrometrica* was separate from the other two mosses. Since the major factor for the edge location was a predominant cell-wall composition of PGA, our results suggested that the chemical component in bryophyte cell wall is a PGA-enriched material.

### Growth performance of different species in excess Pb

**Fig 5:**
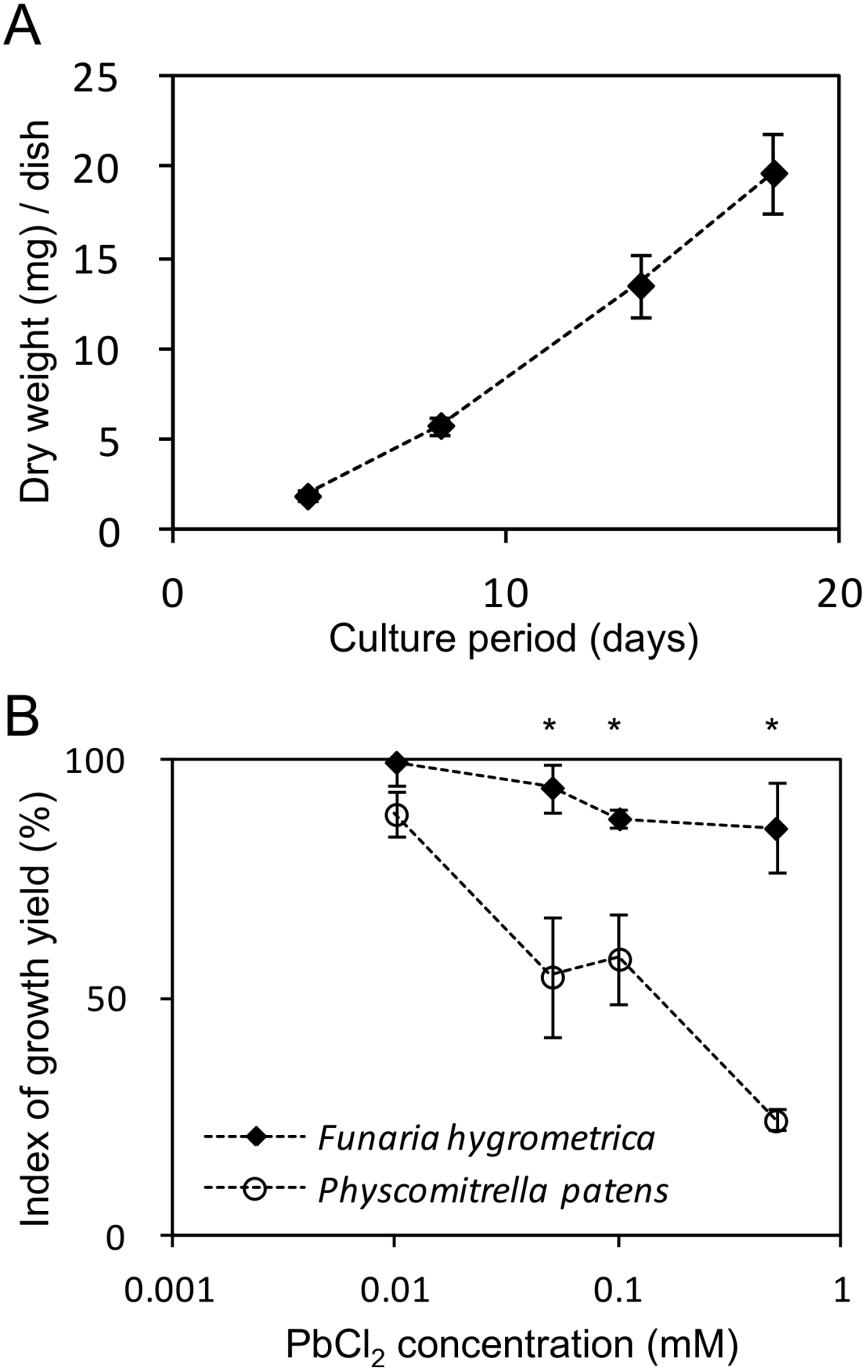
Effect of Pb on moss growth in culture. (A) Growth of *F*. *hygrometrica* on modified Knop’s-agar medium containing 0.5 mM PbCl_2_. At each time point, the dry weight was measured. (B) Effect of PbCl_2_ on the growth yield of *F*. *hygrometrica* and *P*. *patens* at 10 days after sowing. Protonemal cells of *F*. *hygrometrica* and *P*. *patens* were grown on modified Knop’s-agar medium containing various concentrations of PbCl_2_. Index of growth yield [%] = (B/A) × 100, where A is the average weight of the control, and B is the weight of the PbCl_2_-treated sample. Error bars represent the standard deviation of three biological replicates. Measurements marked with asterisks differ significantly as assessed by Welch’s t-test at *p* <0.05.

We examined the viability and growth potential of *F*. *hygrometrica* in the presence of Pb. Even in modified Knop’s medium containing 0.5 mM PbCl_2_, *F*. *hygrometrica* protonemal cells grew (Fig 5A). To see whether other species are similarly tolerant, we compared the growth yields of *F*. *hygrometrica* and *P*. *patens* in the presence Pb concentrations varying from 0.001 to 1 mM (Fig 5B). Both species belong to the *Funariaceae* family, and *P*. *patens* is an established model species for studies in plant evolution and development [32]. At 0.5 mM PbCl_2_, *F*. *hygrometrica* maintained about 80% of its growth yield compared with that observed under Pb-free control conditions, whereas the growth yield of *P*. *patens* was significantly less in 0.05 mM PbCl_2_ and almost completely inhibited at 0.5 mM PbCl_2_. In addition, when we compared the chlorophyll content of *F*. *hygrometrica* and *P*. *patens* grown under a range of Pb concentrations, the chlorophyll *a* and *b* levels of *F*. *hygrometrica* changed little with increasing Pb, whereas those of *P*. *patens* decreased significantly (S2 Table). These results indicate that *F*. *hygrometrica* is a Pb-tolerant species.

### Effect of pH on adsorption

**Fig 6:**
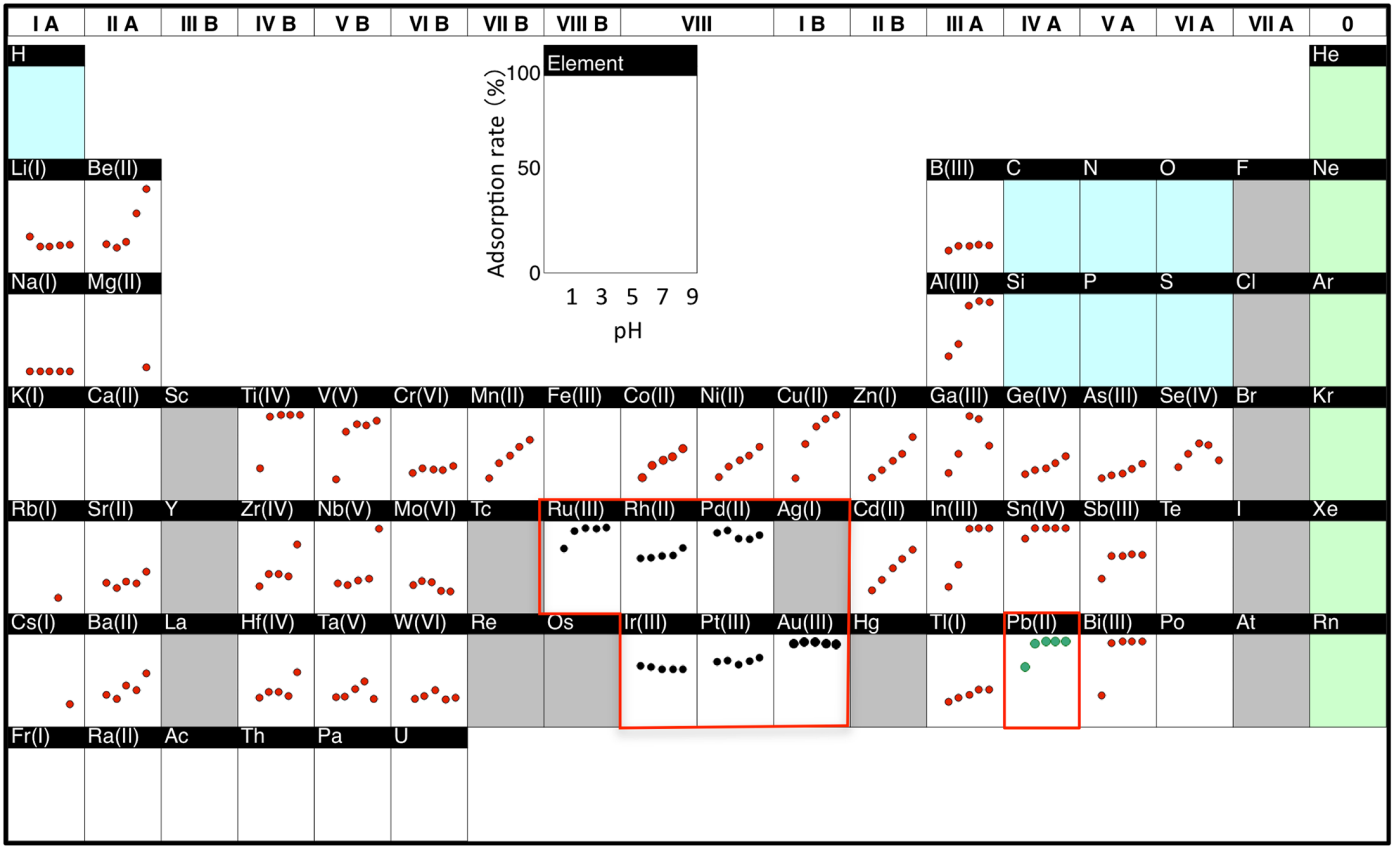
Effects of pH on metal adsorption. *F*. *hygrometrica* protonemal cells were incubated with the metal solutions at the indicated pH values, and the unbound metals in the filtrates were quantified. Adsorption rate (%) = (initial concentration − final concentration) / initial concentration × 100.

To investigate the effects of chemical factors on metal adsorption and desorption, we prepared protonemal cells on a large scale and first examined the dependence of adsorption on pH. Protonemal cells growing in bottles on a shaking incubator were treated with 40 metals including Pb at various pH values, and metal adsorption in the recovered cells was analyzed. The protonemal cells showed various pH dependency patterns for metal adsorption (Fig 6). Among them, adsorption of Pb and Sn was high and stable from pH 3 to 9, whereas adsorption of some other metals, such as Cu, decreased gradually with lower pH, indicating that the Pb adsorption ability was retained in a wide range of pH values. In addition to Pb, the protonemal efficiently adsorbed platinum-group metals (Ru(III), Rh(II), Pd(II), Ir(III), Pt(III)) and Au(III) within a wide pH range.

### Effect of ionic strength on desorption

We further examined the ability to retain Pb in the same system used for monitoring pH effects. When Pb-loaded *F*. *hygrometrica* protonemal cells were incubated in solutions of varying ionic strengths, Pb was stably retained in the cell fraction. More than 95% of the Pb remained adsorbed to protonemal cells, even at 0.5 mol kg^-1^ NaCl (S3 Fig). This result suggests that the bound Pb is not readily desorbed, even under high ionic strength conditions.

## Discussion

In this study, we found that *F*. *hygrometrica* has a remarkable ability to tolerate and accumulate Pb and characterized some physicochemical aspects of metal accumulation by this moss. Our findings led us to propose the use of *F*. *hygrometrica* as a biomaterial for the bio-sorbent filtration of metals. Extensive efforts are now being made to identify and characterize key genes involved in heavy metal uptake and resistance, mainly in vascular plants [33–38]. Our study suggests that bryophytes should be included as target species in these studies.

Our analysis indicated that the cell wall is the main compartment for Pb adsorption; over 80% of the total absorbed Pb was found in the wall (S1 Table). Furthermore, prepared cell-wall fractions adsorbed Pb (Fig 3), suggesting that the chemical constituents and structure of *F*. *hygrometrica* cell walls are responsible for the specificity and large capacity of Pb-accumulation.

Our NMR analyses suggest that the chemical constituents of *F*. *hygrometrica* cell walls are similar to other mosses but distinct from land plants and seaweeds and that PGA is a major component of bryophyte cell walls (Fig 4). At present, it is difficult to identify the chemical properties underlying the hyper-accumulation of Pb in *F*. *hygrometrica*. However, in our previous study, we found that commercially available PGA was as effective in adsorbing divalent Pb as a well-known metal adsorbent, chitosan [39]. Therefore, PGA in the cell walls of *F*. *hygrometrica* might be involved in Pb binding. Although PGA is known to be a major component of pectin as homogalacturonan, NMR analysis did not detect two other major components of pectin, rhamnogalacturonan-I and II. These results suggest that (1) *F*. *hygrometrica* cell walls do not contain typical pectins and (2) this unusual cell-wall composition and structure might be involved in the ability of *F*. *hygrometrica* to adsorb Pb.

Although the mechanism underlying Pb hyper-accumulation in *F*. *hygrometrica* cell walls has not been elucidated, accumulation of heavy metals in cell walls has been reported for other moss species. In *S*. *cataractae*, Satake et al. found that Cu accumulated in the cell wall [40], and Konno et al. suggested that this Cu was bound mainly to pectin [41]. In general, pectin has a high potential for binding divalent ions [42]. For instance, the crosslinking of pectinates by Ca^2+^ ions plays an important role in the organization of polysaccharides in plant cell walls, according to the so-called ‘egg box model’ of pectin structure [43]. Thus, Pb ions may bind to negatively charged polysaccharides such as those found in pectin in *F*. *hygrometrica* cell walls. Comparative biological and physicochemical studies using these mosses are necessary to elucidate the mechanisms for metal specificity and the large capacity for Pb adsorption.

Alternative Pb-binding components in *F*. *hygrometrica* cell walls are the neutral cellulose matrix and/or other constituents with negatively charged moieties such as phosphate groups, carboxyl groups, amines, and amides [44]. Plant cell walls are composed of a complex matrix, whose structure is very diverse. Perhaps *F*. *hygrometrica* has a specialized cell-wall structure for containment of specific metals, or multiple chemical and structural properties might be additively involved in *F*. *hygrometrica’s* tolerance and accumulation of Pb. Riaz et al. reported that the main functional groups involved in Pb adsorption by waste biomass from *Gossypium hirsutum* (cotton) were carboxyl, carbonyl, amino, and alcoholic groups [45].

In our experimental conditions, the thickening of cell walls in the presence of high Pb concentrations was observed *in vivo* and *in vitro* (S2 Fig and Fig 3). Although we cannot provide an unequivocal explanation for the volume enlargement caused by capture of Pb, an extreme structural change could occur in the cell-wall matrix resulting from metal binding. It might be caused by replacement of cell-wall bound Ca^2+^ by Pb^2+^ as previously suggested [46, 47].

In our TEM-EDX analysis, Pb was detected not only in cell walls but also in chloroplasts and ER-like structures (Fig 2). Given that *F*. *hygrometrica* can grow under high PbCl_2_ concentrations, it is plausible that *F*. *hygrometrica* has a Pb detoxification mechanism. For example, the Pb detected in chloroplasts and ER might be detoxified by sequestration with chelating compounds or proteins and excretion to the extracellular space or vacuole. Elucidation of the detoxification mechanism will be important for understanding the specialized competence of this moss in metal tolerance and accumulation.

Our study also revealed the efficient adsorption of other metals, such as Sn, Au and Pt-group metals, by *F*. *hygrometrica* protonema under a wide pH range (Fig 6). At present, it is not clear whether there is a common adsorption mechanism for these metals with that for Pb. Interestingly, under the same experimental conditions, the binding properties of the protonemal cells to Pb(II), Au(III), Ru(III), Rh(II), Pd(II) appear comparable or superior to that of a commercial chelating resin that immobilizes ethylenediaminetriacetic acid and iminodiacetic acid groups [48], tempting us to test the use of the moss for recovery of other metals.

Our results suggest that *F*. *hygrometrica* is a useful bio-material for the recovery of heavy metals, especially Pb from aqueous solutions. Currently, metal ions are recovered by chemical sedimentation, electro deposition, or ion-exchange adsorption, using materials and energy ultimately derived from fossil resources. Although current methodologies work well, alternative technologies based on plant-derived materials will become more important in the future. The application of mosses for Pb-recovery from industrial wastewaters should be valuable for creating a sustainable recycling system.

## Supporting information

S1 Fig Column test of *F*. *hygrometrica* protonemal cells. (A) Schematic diagram of the column-test procedure for analyzing the metal adsorption capacity of the moss *F*. *hygrometrica*. Fh, *Funaria hygrometrica*; MQW, MilliQ water. (B) Adsorption of Pb and Au to *F*. *hygrometrica* protonemal cells. PbCl_2_ or AuCl solution was loaded onto Fh-columns. The filtrates were collected and analyzed by ICP-MS. Relative concentration in filtrate (%) = actual filtrate concentration / initial concentration × 100.
(TIFF)

S2 Fig Effect of PbCl_2_ treatment on cell wall thickness of *F*. *hygrometrica* protonemal cells. After treatment of protonemal cells with (+) or without (-) 100 μM PbCl_2_ in a column test, the cells were fixed. Cross-sections of protonemal cells including basal cells were photographed with a Gatan DualView CCD camera. Widths of cell walls were measured using PhotoMeasure Version 2.20 (Kenis Co., Japan). Error bars represent the standard deviation of six biological replicates. The two samples differed significantly as assessed by Welch’s t-test at *p* <0.02.
(TIFF)

S3 Fig Effect of ionic strength on Pb desorption. Pb-adsorbing *F*. *hygrometrica* protonemal cells were incubated at the indicated ionic strengths, and the released Pb in the filtrates was quantified. Retention rate (%) = (initial Pb amount − desorbed Pb amount) / initial Pb amount × 100.
(TIFF)

S1 Table Distribution of Pb in *F*. *hygrometrica* protonemal cells. In this analysis, [Pb]_CWF_ was 82.0 mg g^-1^ dry weight and [Pb]_TC_ was 56.6 mg g^-1^ dry weight. CWF/TC was 61.2%. [Pb]_CW_ was 50.2 mg g^-1^ dry weight.
(TIFF)

S2 Table Chlorophyll content of protonemal cells exposed to different PbCl_2_ concentrations in *F*. *hygrometrica* and *P*. *patens*. *F*. *hygrometrica* and *P*. *patens* protonemal cells were cultured in modified Knop’s liquid media containing the indicated concentrations of PbCl_2_ for 10 days. Thirty mg of freeze-dried samples were used to measure the chlorophyll concentration as described by Arnon (1949). Chl a, chlorophyll *a*; Chl b, chlorophyll *b*; NI, no inhibition; ND, not determined. ^*a*^, Relative Inhibition (RI, %) was calculated as RI = (1 –A/B) × 100, where A is the average value determined in the control (0 mM PbCl_2_) and B is the average for the treatment. *, Significant difference as assessed by Welch’s t-test at *p* <0.05 (n = 3).
(TIFF)
