# Supplementary figures and images for: Protonema of the moss Funaria hygrometrica can function as a lead (Pb) adsorbent
Source: PLoS One. 2017 Dec 20;12(12):e0189726. doi: 10.1371/journal.pone.0189726 (PMC5738082; doi:10.1371/journal.pone.0189726)

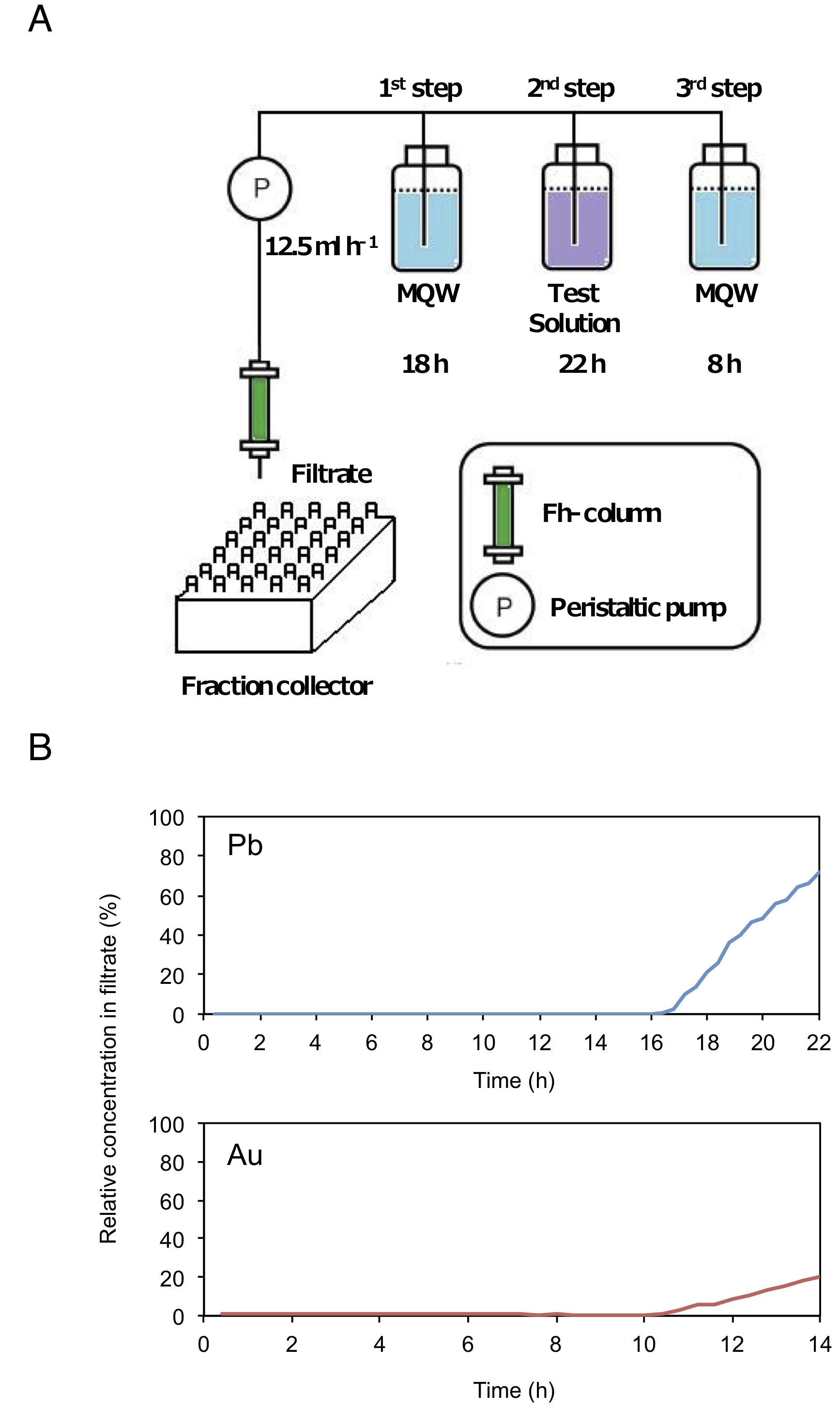

Supplement: S1 Fig — (A) Schematic diagram of the column-test procedure for analyzing the metal adsorption capacity of the moss F. hygrometrica. Fh, Funaria hygrometrica; MQW, MilliQ water. (B) Adsorption of Pb and Au to F. hygrometrica protonemal cells. PbCl2 or AuCl solution was loaded onto Fh-columns. The filtrates were collected and analyzed by ICP-MS. Relative concentration in filtrate (%) = actual filtrate concentration / initial concentration × 100. (TIFF) [file pone.0189726.s001.tiff]

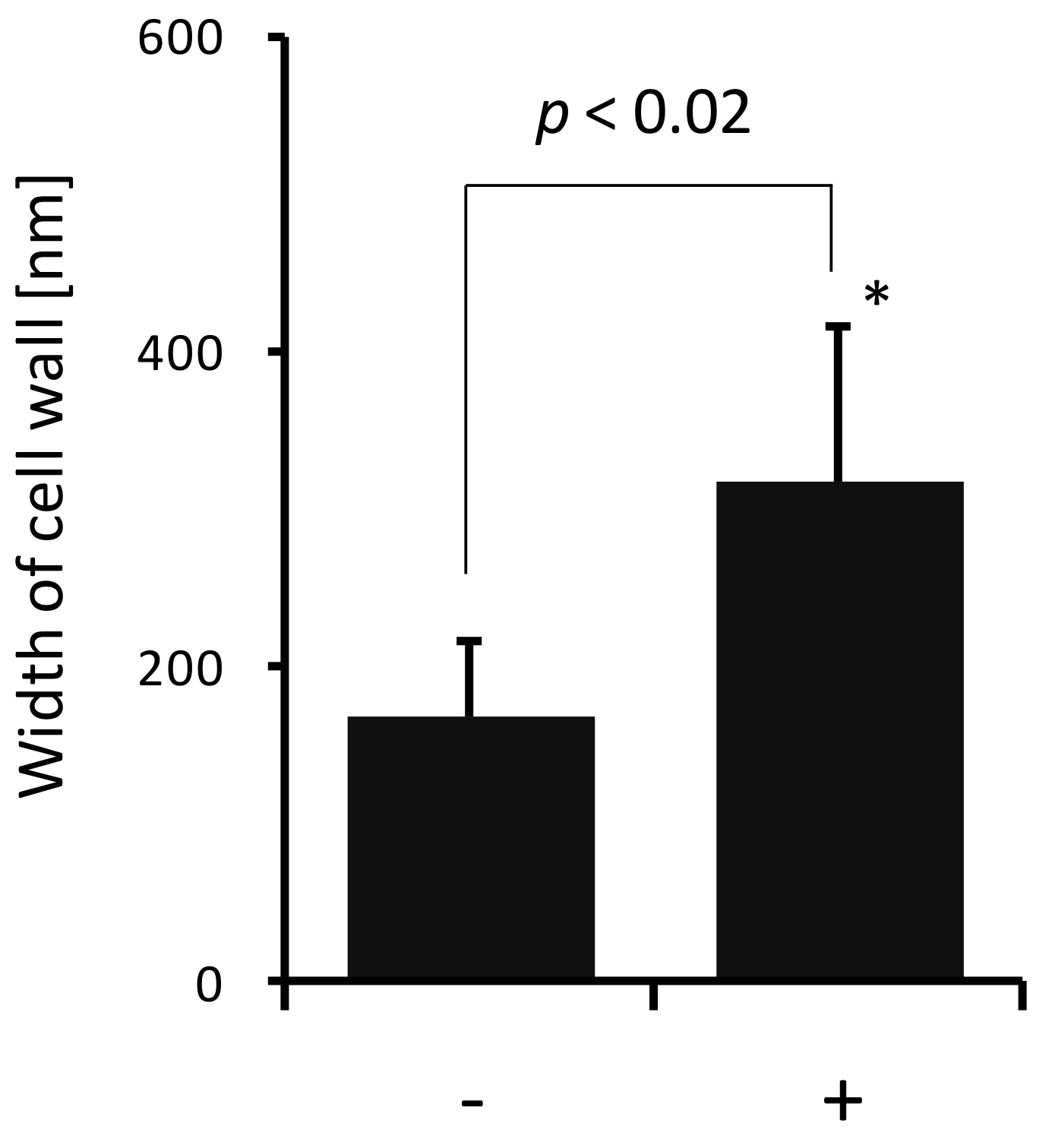

Supplement: S2 Fig — After treatment of protonemal cells with (+) or without (-) 100 μM PbCl2 in a column test, the cells were fixed. Cross-sections of protonemal cells including basal cells were photographed with a Gatan DualView CCD camera. Widths of cell walls were measured using PhotoMeasure Version 2.20 (Kenis Co., Japan). Error bars represent the standard deviation of six biological replicates. The two samples differed significantly as assessed by Welch’s t-test at p <0.02. (TIFF) [file pone.0189726.s002.tiff]

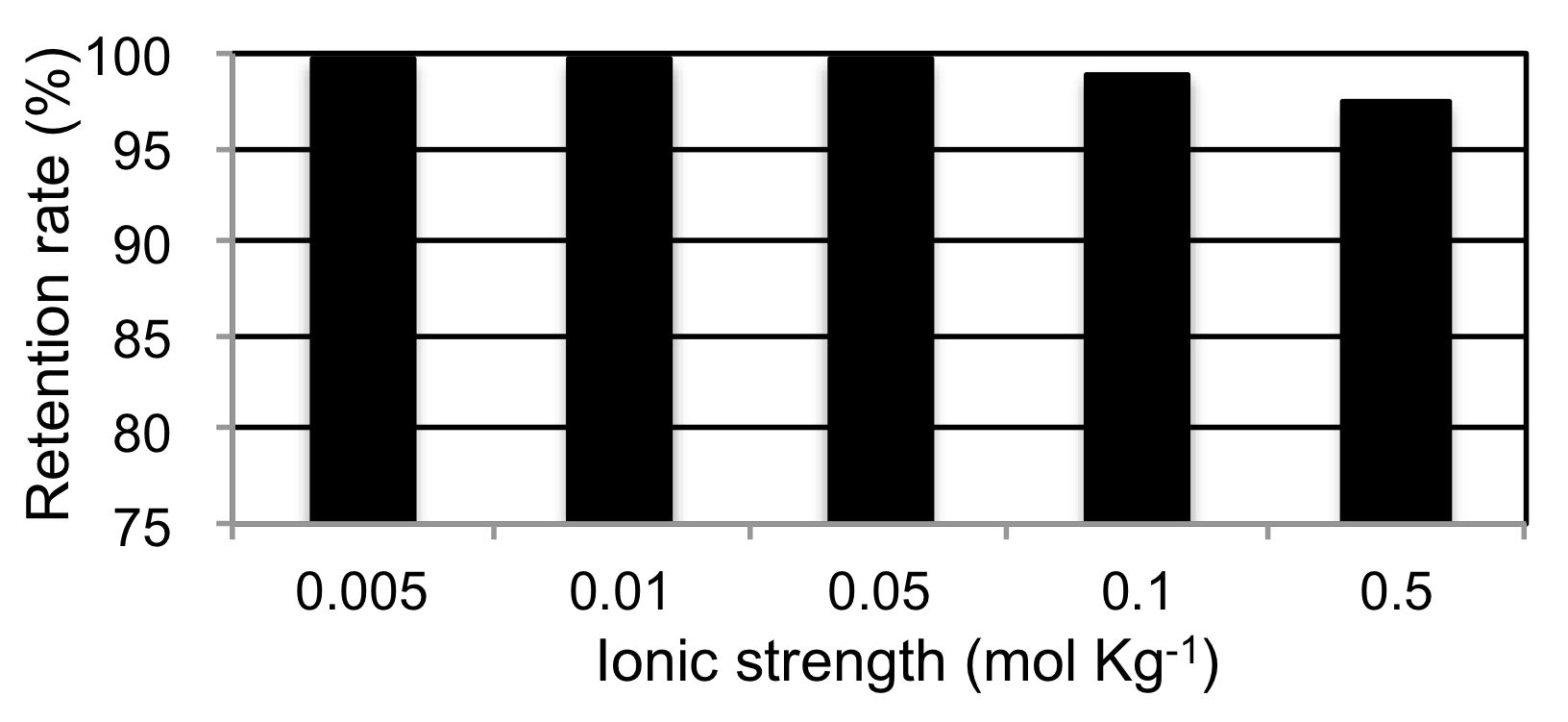

Supplement: S3 Fig — Pb-adsorbing F. hygrometrica protonemal cells were incubated at the indicated ionic strengths, and the released Pb in the filtrates was quantified. Retention rate (%) = (initial Pb amount − desorbed Pb amount) / initial Pb amount × 100. (TIFF) [file pone.0189726.s003.tiff]

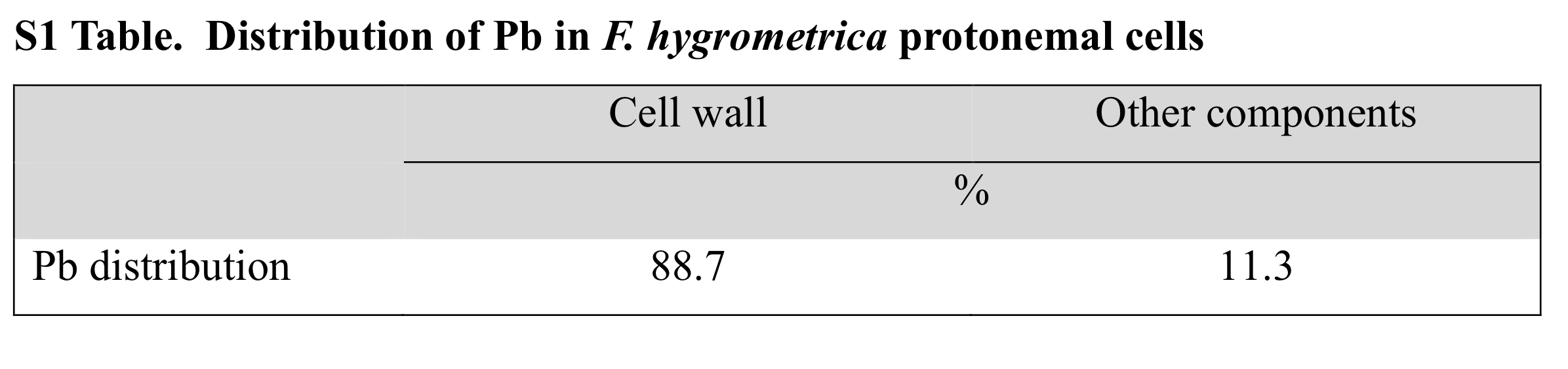

Supplement: S1 Table — In this analysis, [Pb]CWF was 82.0 mg g-1 dry weight and [Pb]TC was 56.6 mg g-1 dry weight. CWF/TC was 61.2%. [Pb]CW was 50.2 mg g-1 dry weight. (TIFF) [file pone.0189726.s004.tiff]

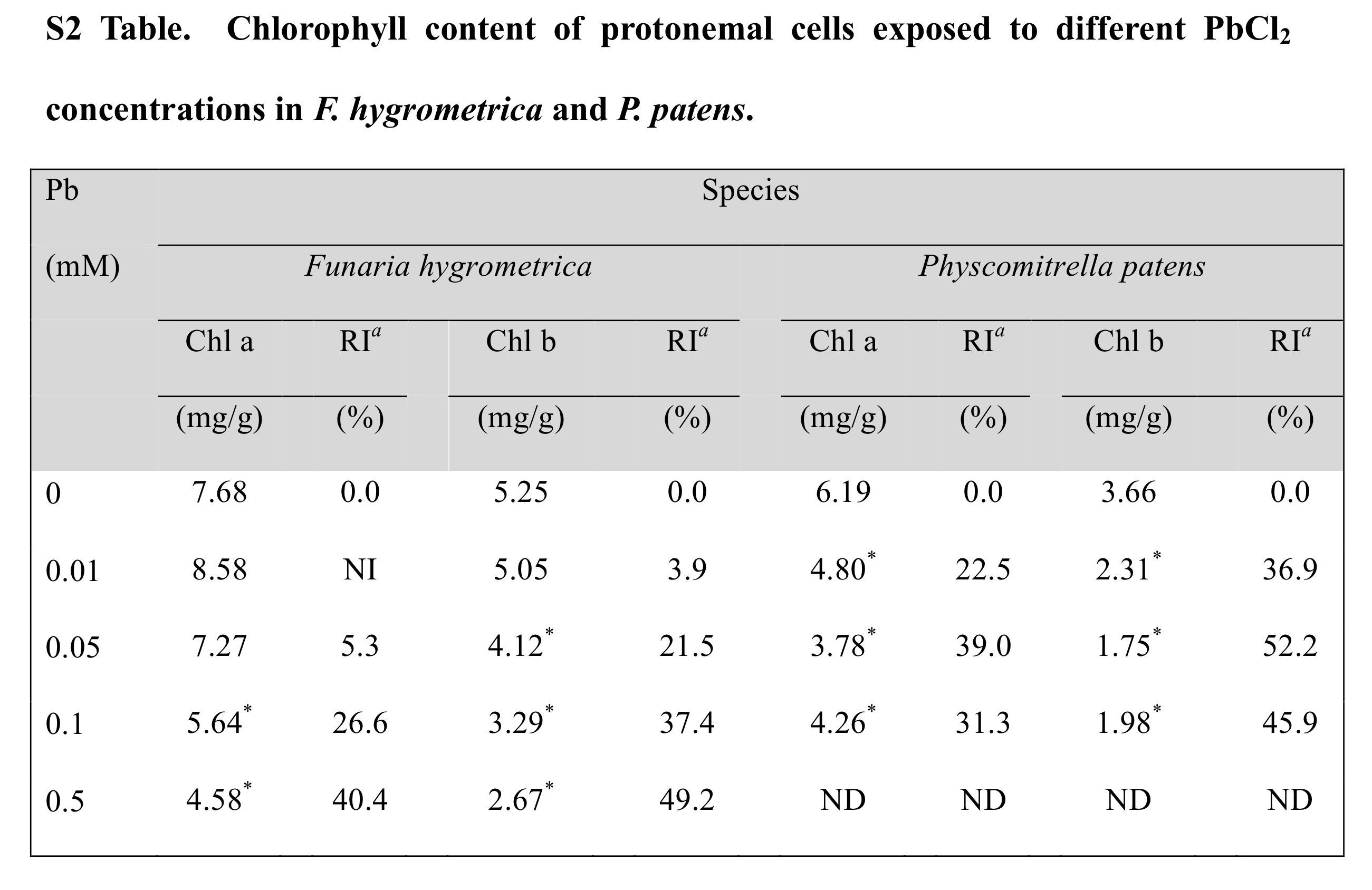

Supplement: S2 Table — F. hygrometrica and P. patens protonemal cells were cultured in modified Knop’s liquid media containing the indicated concentrations of PbCl2 for 10 days. Thirty mg of freeze-dried samples were used to measure the chlorophyll concentration as described by Arnon (1949). Chl a, chlorophyll a; Chl b, chlorophyll b; NI, no inhibition; ND, not determined. a, Relative Inhibition (RI, %) was calculated as RI = (1 –A/B) × 100, where A is the average value determined in the control (0 mM PbCl2) and B is the average for the treatment. *, Significant difference as assessed by Welch’s t-test at p <0.05 (n = 3). (TIFF) [file pone.0189726.s005.tiff]
